# Implementation of a crisis resolution team service improvement programme: a qualitative study of the critical ingredients for success

**DOI:** 10.1186/s13033-024-00638-6

**Published:** 2024-05-04

**Authors:** Danielle Lamb, Alyssa Milton, Rebecca Forsyth, Brynmor Lloyd-Evans, Syeda Akther, Kate Fullarton, Puffin O’Hanlon, Sonia Johnson, Nicola Morant

**Affiliations:** 1 Department of Applied Health Research, UCL, Gower Street, London, WC1E 6BT UK; 2 Faculty of Medicine and Health, The University of Sydney, Sydney, Australia; 3 Division of Psychiatry, UCL, London, UK

## Abstract

**Background:**

Crisis Resolution Teams (CRTs) offer home-based care for people in mental health crisis, as an alternative to hospital admission. The success of CRTs in England has been variable. In response to this, the CRT Optimization and RElapse prevention (CORE) study developed and trialled a 12-month Service Improvement Programme (SIP) based on a fidelity model. This paper describes a qualitative evaluation of the perspectives of CRT staff, managers, and programme facilitators. We identify barriers and facilitators to implementation, and mechanisms by which service improvements took place.

**Methods:**

Managers and staff from six purposively sampled CRTs were interviewed, as well as six facilitators who were employed to support the implementation of service improvement plans. Semi-structured focus groups and individual interviews were conducted and analysed using thematic analysis.

**Findings:**

A majority of participants viewed all components of the SIP as helpful in improving practice, although online resources were under-used. Perceived barriers to implementation centred principally around lack of staff time and ownership. Support from both senior staff and facilitators was essential in enabling teams to undertake the work associated with the SIP. All participating stakeholder groups reported that using the fidelity model to benchmark their CRT work to best practice and feel part of a ‘bigger whole’ was valuable.

**Conclusion:**

CRT staff, managers and programme facilitators thought that a structured service improvement programme helped to increase fidelity to a best practice model. Flexibility (from all stakeholders) was key to enable service improvement actions to be manageable within time- and resource-poor teams.

**Supplementary Information:**

The online version contains supplementary material available at 10.1186/s13033-024-00638-6.

## Background

Crisis Resolution Teams (CRTs) are multidisciplinary mental health teams that provide rapid assessment and short-term intensive treatment to people in the community during a mental health crisis [1, 2]. Also known as ‘Crisis Resolution and Home Treatment Teams’, or simply ‘Home Treatment Teams’, their aim is to avoid admission of service users to psychiatric inpatient wards, or facilitate early discharge from hospital through their gatekeeping function if admission is deemed necessary. CRTs were mandated in England by the NHS Plan in 2000 [3] and have been implemented in nearly all local healthcare organisations (known as ‘Trusts’) in England [4, 5]. The model was developed in the USA and Australia, and have been implemented nationally in England and Norway [6]. Trial evidence [7, 8] and observational studies [9] suggest CRTs can reduce inpatient admissions and improve service user satisfaction, although results relating to satisfaction are mixed [10, 11].

Following the widespread implementation of CRTs in England, wide variations in how they function have been found, arguably related to the lack of a clearly defined CRT model [5]. In response, an evidence-based CRT fidelity scale was developed to measure the extent to which CRTs operated according to a model of best practice [12]. This enables CRTs to take actionable steps to improve their service delivery. The CRT fidelity scale consists of 39 items outlining best practice in areas such as responding quickly to new referrals, providing individualised care, having a multidisciplinary team, and working effectively with other services. Subsequently, the scale has been used in the UK and Norway for service evaluation purposes and to benchmark CRT practice nationally. However, results show that CRTs have typically achieved only low or moderate fidelity to this best practice model (Hasselberg et al., 2021; Lloyd-Evans et al., 2017).

A critical barrier to widespread use of evidence-based practices may be lack of knowledge and resources about how to implement service-level changes [13]. To address this challenge, the CRT Optimisation and Relapse Prevention (CORE) study developed a CRT service improvement programme (SIP), that aimed to improve fidelity to the CRT best practice model by encouraging service changes and improvements in relevant domains. The impact of this service-level intervention was subsequently evaluated in a cluster-randomised trial [14, 15], which found that the 12-month SIP was effective in improving teams’ model fidelity and reducing hospital admissions. There have been recent calls for research into the barriers, facilitators, and implementation processes of systematic approaches to aligning service delivery with evidence-based best practice models in order to enhance understanding of their critical ingredients, and to improve their impact, uptake and sustainability [16]. The current paper reports on a qualitative study embedded within the CORE SIP trial.

We aim to understand CRT managers’ and clinicians’, and programme facilitators’ experiences of the SIP, and to identify barriers and facilitators to implementing the service improvement programme, and the critical ingredients or mechanisms by which improvements were achieved.

## Methods

### Ethical approval

for the study was granted by Camden & Islington Research Ethics Committee (Ref: 14/LO/0107). The trial of the intervention discussed in this paper is registered on the ISRCTN registry (Ref: 47,185,233). The study was carried out in accordance with the Declaration of Helsinki.

### Study context: the CORE Service Improvement Programme (SIP) trial

Within the CORE cluster-randomised trial of the CRT SIP [14, 15], fifteen of twenty five CRTs in England took part in a 12-month service improvement intervention in 2014–2015. This work drew on the Implementing Evidence Based Practice approach (Drake et al. 2003), which provides a framework for implementing complex service-level interventions using fidelity measurements and service improvement resources to help teams move towards an evidence-based model of best practice. The development of a 39 item fidelity measure was based on multiple sources of evidence about CRT good practice including a systematic review [2], a national CRT survey [17], and qualitative interviews with stakeholders [11] (i.e. CRT staff and managers, clinicians referring service users to CRTs, and service users, and carers). The multicomponent CRT service improvement programme provided resources, structures and tailored support to CRTs to encourage teams to improve their scores on this measure over a 12 month period. SIP design was informed by factors identified as contributing to attainment of high fidelity in the USA Implementing Evidence Based Practices Program [18]: ‘prioritisation of the programme’ (understanding and attitudes of those involved, financial/resource provision); ‘leadership support’ (the culture created, responsibility for leading improvement work, engagement with the programme); ‘workforce development’ (sufficient staffing, training and supervision); ‘workflow re-engineering’ (staff meetings, documentation, policies); and ‘practice reinforcement’ (outcome monitoring, fidelity, feedback). Components of the SIP included online resources, a scoping day, learning events, coaching by facilitators (an experienced clinician or manager who supported implementation), fidelity reviews, routine meetings, and research team support. These are described in more detail in Table 1.

**Table 1:** CRT service improvement programme core components

Components	Description	Timing
Fidelity reviews	A team of three reviewers (a clinician, a peer researcher, and an academic researcher) visited a CRT for one day at the start of the intervention period, at 6 months, and at 12 months. They conducted interviews (with the manager, staff, other services, service users, and carers) and reviewed documentation (policies and operating procedures, anonymised case notes) to assess the team’s adherence to a model of good practice as indicated by 39 items of the CRT fidelity scale. After the visit, the CRT received a report outlining areas of positive practice and areas to consider for service improvement, with reference to each item in the CRT fidelity scale.	Start of the programme, at 6 months, and at 12 months (end of the programme)
Scoping day	A ‘scoping day’ was delivered with the whole team for them to jointly prioritise and plan service improvement goals. Using the fidelity review report, the facilitator, CRT manager, and CRT staff identified areas they wanted to develop, and what actions they could take to address identified areas of need.	Early in the programme, ideally within the first month
Multi-team learning events	Two face-to-face learning events, one day in duration, where staff from CRTs implementing the SIP in the CORE trial met each other, facilitators, and other experts. Events consisted of discussions and workshops about team functioning based on fidelity reviews, and sharing of good practice.	One at 5 months and one at 9 months
Online resources	A freely accessible website with pages for each CRT fidelity scale item, including the criteria for that item, as well as examples of good practice, case studies, links to relevant research, reports, and other websites, example leaflets and document templates, and recordings of interviews with experts.	Available throughout
Routine meetings	Regular meetings of a CRT management group and the facilitator were held throughout the 12-month period to develop and review detailed service improvement plans.	Once per month
Coaching by facilitators	Coaching from an experienced clinician or manager who was employed as a facilitator half a day per week to offer the CRT manager and staff advice and support with developing and implementing service improvement plans. Facilitators were typically from within the same healthcare organisation as the CRT, sometimes within the CRT, though one facilitator was external to the organisations they supported.	Available throughout
Actions	SIPs included actions such as small groups of staff re-drafting forms or documentation their team and service users regularly used, involving service users and carers in e.g. staff recruitment, agreeing new or redefined policies for CRT systems and processes internally, improving communication with teams referring to, or being discharged to from, the CRT.	Occurred throughout
Research team support	The research team had regular contact with each facilitator (including supervision sessions provided by a clinical psychologist), collected service improvement plans from each team, and coordinated and conducted the three fidelity reviews.	Available throughout

### Sampling

This study focussed on six sites that were purposively selected from the 15 teams receiving the service improvement intervention in the CORE trial. Site selection was carried out following the 12-month fidelity reviews to include variations on three criteria: setting (teams in urban and more rural settings); starting points (teams with comparatively high and low model fidelity scores at baseline); and change during the SIP (teams where a large improvement in the fidelity score was achieved, and where it was not).

### Participants and recruitment

All facilitators who had worked with the selected CRTs and managers of all selected CRTs were contacted by members of the CORE team by phone or email after the end of the 12-month intervention period, inviting them to participate in individual interviews. All staff who had been working in the team during the intervention period were invited via their team manager (who were contacted by members of the CORE team by phone or email) to attend a focus group. All potential participants were given an information sheet about the study, and provided informed consent before participation. Additional focus groups were held in teams where not all participants were able to meet at the same time, e.g. due to shift work. It was made clear that participation was voluntary, and that findings would be anonymised. We use the term ‘stakeholders’ to refer to the groups participating in this study (i.e. CRT managers, CRT staff, and facilitators), but recognise that this does not include service users or carers, so the term should not be taken to imply all stakeholders.

### Data collection

Semi-structured interview and focus group schedules were designed for use with each group of participants (facilitators, managers, and staff), with input from professionals working in CRTs and other mental health services, as well as from people who had used such services. This aimed to ensure that we addressed areas important to multiple different groups of stakeholders. Questions included participants’ views on: the SIP overall; specific elements of the SIP (fidelity reviews, the online resources, the facilitator etc.); what had been helpful or unhelpful during implementation; and the impact the SIP had on individual staff members, and within the CRT as a team (see Appendix 1 for example topic guides). In addition, facilitators were asked about their experiences of facilitating the SIP, and barriers and facilitators of SIP implementation whilst in their role.

Face to face interviews and focus groups were conducted by two researchers from the CORE team (DL, KF, PO), at locations convenient to the participants (e.g. facilitators’ offices and CRT sites). Data was audio recorded and transcribed by an external agency. Transcripts were checked for accuracy and anonymity by the research team.

The interviewers were all white women in their late 20s and early 30s, with between five and 10 years of research experience, all of whom had personal experience of either using mental health services themselves and/or supporting loved ones who used mental health services. The wider research team contributing to this paper include older and younger male and female researchers, of White and Asian ethnicity, some of whom have worked clinically in CRTs and other mental health services. The inclusion of researchers with diverse personal characteristics and experiences enabled multiple perspectives to be considered.

### Analysis

Data were analysed using thematic analysis [19, 20] within NVivo software. We adopted an approach that combined an inductive stance with a focus on our initial broad research questions about participants’ experiences and views about implementation of the SIP. Preliminary codes were developed using a selected sample of data from each stakeholder group. These were iteratively expanded and refined as more data was coded, with continuous conceptual reviews to group codes into meaningful topic domains and themes. Comparisons between data from each stakeholder group (CRT staff, managers and SIP facilitators) were made throughout the analysis process to explore both similarities and variations. We also compared between CRTs in order to explore variations in SIP implementation and how these might relate to different outcomes and contexts. Analysis was collaborative throughout: DL, RF, SA, KF, and PO coded the data, and discussed and iteratively refined the coding framework with NM, BLE, SJ, and AM. This enhanced validity by ensuring that analysis was comprehensive and conceptually coherent and by encouraging a reflexive stance [21]. The team consisted of clinical and non-clinical researchers working for and leading the CORE study, some of whom had contributed to development of the SIP although none had been involved in its delivery.

## Findings

### Participating sites and individuals

We recruited six sites for this study as intended, however, two originally selected CRTs met challenges when implementing the SIP and declined to take part in this study, so two alternative CRTs were selected. Details of the six participating CRTs and their engagement with the SIP intervention are presented in Table 2. The descriptions of each CRT’s engagement with the SIP were collated from reflexive field notes taken by researchers during the course of the intervention, during and after visits to each team.

Individual interviews were carried out with facilitators (*n* = 6) and with the managers of each of the teams (*n* = 8; in two sites two people had managed the teams during the 12-month SIP). Eight interviews were carried out with a total of 16 CRT staff who had worked in the participating teams during the 12-month intervention period, with one to three people attending each interview.

**Table 2:** CRT characteristics and SIP engagement

Team and sampling criteria: a) Location b) Baseline fidelity score c) Small or large fidelity score change	Fidelity measure change (baseline to post intervention; 12 months)*	Description of each CRT’s engagement with Service Improvement Programme (SIP)	Fidelity items and domains targeted	SIP components completed					
				Scoping day	Learning events	Regular coaching	Fidelity reviews	Routine meetings	Research team support
1 a) Rural b) Low fidelity c) Large change	105 to 123 (+ 18)	The team manager was sceptical about CORE, which affected staff engagement. In months 1–3, the facilitator visited the team 4 times to outline plans and formulate SIPs. The facilitator did lots of the work herself, and found it difficult to keep on track of work the team was doing. The interim fidelity review took place in month 7. The Team away day took place in month 8, with the facilitator focusing on engaging staff. Reflection on progress took place in month 14.	**8** Interface with CMHRS & EIP teams **11, 13, 14** Work with carers **16** Monitoring & information on medication side effects **17** Access to psychological therapies	Yes	No	Yes	Yes	No	
2 a) Urban b) Low fidelity c) Moderate change	98 to 111 (+ 13)	Communication with the newly appointed manager was difficult throughout. Staff didn’t know much about the study, SIP structures or processes. The facilitator was minimally involved. The team was very busy, and had high staff turnover. Potential areas for service improvement were established in month 2. In month 4, the baseline fidelity report was reviewed. In month 6, the scoping day took place. The facilitator reviewed progress in month 7.	**2** CRT’s accessibility to eligible referrers **7** Facilitating early discharge **11** Comprehensive assessment **24** Relapse prevention planning **30** Staff induction/ongoing training **36** Named worked system	Yes	No	No	Yes	No	Yes
3 a) Suburban b) Moderate fidelity c) Large change	130 to 149 (+ 19)	The facilitator was very engaged and enthusiastic. Once in post, the returning manager was also engaged. Staff followed SIP structures and processes, with small working groups meeting regularly. The facilitator visited the team at least fortnightly, working with all staff. Despite efforts, the facilitator reported feeling that CORE was not fully embedded into the team. The interim review took place in month 8. In month 13, progress made was reviewed and ongoing work was discussed.	**11** Assertive & comprehensive assessment **14** Carer support **16** Medication communication **32** Lone working policy	Yes	Yes	Yes	Yes	Yes	Yes
4 a) Suburban b) Moderate fidelity c) Small change	138 to 142 (+ 4)	The team was well-established with an engaged manager. A bi-monthly working group of senior staff was established in month 1. The facilitator didn’t visit the team every week. Service improvement plans were discussed at the scoping day in month 4. The interim fidelity review took place in month 9. The team reported struggling to embed newly developed resources during the intervention period, but were interested in continuing with service improvement work.	**11** Initial assessment **12** Information for service users and families **14** Work with carers **24** Relapse prevention **30** Supervision **36** Named workers	Yes	Yes	Yes	Yes	Yes	Yes
5 a) Suburban b) Moderate fidelity c) Large change	133 to 155 (+ 22)	The manager was extremely enthusiastic. Some staff were very engaged, and the team closely followed SIP structures and processes. The facilitator was not based locally; visits were infrequent with little staff interaction. The team was always busy, but made time for CORE. The scoping day took place in month 2. The interim fidelity review took place in month 8. The facilitator reported work progressed slowly during the programme.	**4, 24** Planning for future crises **6, 7** Early discharge **8, 25** Aftercare & onward referral **10** Distinct service **12, 21, 38** Timing, length & frequency of visits **13, 14, 16** Support for Sus & families **17, 29** Psychological interventions & staff to deliver **22, 30, 31, 32** Staff recruitment, induction, monitoring & training **34** Working with community services **35** Equality & diversity **36** Staff consistency **37** Alternatives to hospital	Yes	Yes	Yes	No	Yes	Yes
6 a) Rural b) Low fidelity c) Large change	97 to 134 (+ 37)	Before the intervention the team was seen as failing, and a new, enthusiastic manager was appointed. Staff were a mixture of engaged and disengaged. Facilitator visits were not very frequent. The scoping day took place in month 2. Most of the service improvement work was done by the manager. The interim fidelity review took place in month 8, with a revised SIP being agreed upon by all following this. Monthly update meetings took place until the end of the study.	**7** Early discharge **8** Resource lending library **15, 16** Medication & prescribing **20** CRT integrated care pathways **27, 28, 29, 30** Staffing levels **30** Skill mapping, role/staff development **34, 35** Communication & boundaries	Yes	No	No	Yes	No	Yes

*Total scale range = 39–195. Scores below 117 were considered low fidelity; scores of 117–155 were moderate fidelity; 156 and above were high fidelity [12]

### Qualitative findings

We present findings below in three sections that describe three broad topic domains in our data: (i) respondents’ views about components of the 12 month service improvement programme; (ii) process factors that highlight perceived barriers and facilitators to SIP implementation; and (iii) perceived impacts of the programme (Table 3).

**Table 3:** Themes and Subthemes

Theme	Sub Theme
Views on components of the SIP	Fidelity reviews
	Scoping day and events
	Online resources
	Facilitator support
Process factors (perceived barriers and facilitators to implementation)	Resources, staff and time
	Staff engagement and buy-in
	SIP as a vehicle for change
	Culture of support from the top
	The value of service user input
Perceived impact of the SIP	Motivation and purpose
	Team and inter-service communication
	Sustainable structural service improvements
	Low level of SIP impact

## Components of the SIP

### Fidelity reviews and report

Fidelity reviews were frequently described as a time consuming process, requiring substantial preparation from teams. Some participants identified a lack of sensitivity in how far the fidelity could capture the ways CRTs were organised locally. For example, some CRTs had no control over certain elements of the model, resulting in low fidelity scores: “*I got a bit defensive…well actually we’re not commissioned to do that and yet you are measuring us against something we aren’t going to fit.”* (*Manager CRT 1*). The same CRT manager questioned whether reviewers had sufficient understanding of their local CRT context. Overall, however, some participants from every team considered the fidelity review process a beneficial experience. In particular, managers and staff thought reviews encouraged staff motivation and fostered engagement, ownership, team working and reflective practice.

*I think they were useful for the team to reflect together, have someone external come in and ask questions. It made people more aware of the items in the model, the emphasis on the service user feedback. (Facilitator CRT 3).*

Participants described the fidelity review report as providing valuable feedback, especially as it was delivered by a reputable external source. Reports helped teams to plan their approach to quality improvements facilitated teams’ ability to benchmark themselves against a best practice model:

*For me it provided a framework, so I had a written guide of what a good crisis team would look like and something clear to work towards. (Manager CRT 6).*

### Scoping day and CORE learning events

The scoping day was generally viewed as helpful in promoting engagement and action planning, and providing a dedicated time and place for discussion.

*I guess I sort of felt like it brought that passion back in people that, you know, this is what we do and this is why we do it and this is how we could do it better. (Staff CRT 5)*

In particular, the structured agenda of the scoping day was viewed as helpful by facilitators: “*The feedback from the teams was really good and I think that was largely to do with the template we were given*.” (*Facilitator CRT 3*). In some teams, however, there was a delay in holding the scoping day at the beginning of the study, and this time lost within the 12-month programme reduced opportunity for progress.

The additional off-site learning events that followed the scoping day were also viewed positively, but were mentioned less frequently than the scoping day. Some participants valued these opportunities to get away from the team base as a group, particularly to meet other CRTs, compare how they were approaching their SIP work and feel a sense of a collective CRT identity.

### Online resources

Despite participants stating that they had intentions to use the SIP online resources, these were under-used, with only a few participants reporting regular use of the website, mainly for browsing and fact-finding: “*It just helped bolster my own information really, as to understanding it all.” (Staff CRT 3)*. Some did use the SIP website for planning service improvements and reported its benefits.

*So the resource kit, I think, for me, was very, very helpful because you could see what other people were doing, what they were trying to do, and then you can then try and modify those things to your service. (Staff CRT 4).*

### Facilitator support

CRT staff and managers’ experiences of team support provided by facilitators were mainly positive, with most finding their facilitator approachable and helpful, particularly as a sounding board for ideas. Regular facilitator contact with CRTs was viewed as helpful, especially in increasing focus on quality improvement work.

*The two fortnightly meetings that we had with [name of facilitator], they were really, really helpful and that was good…[the] team all getting together and talking about, you know, getting ideas and looking at ways we could all improve. So it felt like rather than being, sort of, dictated to about how ‘this is what we need to be doing’, it was more about involving everybody. (Staff CRT 3).*

In CRTs 5 and 6, managers felt the facilitator lacked presence within the team, and thought a facilitator embedded in the CRT would have better ensured SIP momentum. In CRT 2 the facilitator was also not embedded in their allocated CRT, and felt that this lack of presence made their role slightly ineffectual: “*If I was starting again now …I think I would’ve boundaried that time a lot more strictly.”* (*Facilitator CRT 2)*.

Most teams and facilitators emphasised that it was (or would be) more helpful when facilitators where internal and known to the CRT and the Trust (local healthcare organisation). This promoted engagement and a sense of trust. Pre-existing knowledge of teams and their practice enabled better quality improvement planning, giving more credibility to the role:

*Being part of the Trust was really important to gain the trust of the teams and to gain a bit of credibility. I think if I’d been an outside consultant I don’t think it would have worked. (Facilitator CRT 1)*.

## Process factors (barriers and facilitators to implementation)

### Resources, staff and time

A lack of time to engage with the CORE SIP was the most frequently cited process barrier across all teams: “*That’s the biggest challenge, just finding the time to sift through the things, and actually not being able to do it.”* (*Manager CRT 1)*. The busy and over-stretched nature of providing crisis home treatment was emphasised repeatedly across all stakeholder groups, with comments that 12 months did not give teams enough time to implement service changes and see this take effect:

*I feel like with the amount of improvement, the scope for improvement that there was, a bit more time was required to make significant developments (Manager CRT 6)*.

Related to this, SIP implementation was affected by a lack of staff availability due to : *“ …sickness, annual leave, that sort of thing, people leaving the team.*” (*Staff CRT 6*). This included staff vacancies, and practical difficulties in arranging meetings as a team due to the busy shifts and clinical work. Changes in team leadership were also an important implementation barrier: “*We’ve had lots of changes in management and leadership. So maybe what some people said they would do they weren’t able to do*” (*Staff CRT 6*).

### Staff engagement and buy-in

Team ownership and engagement was mentioned by a majority of stakeholders across multiple CRTs. Lack of team ownership of the SIP appeared, in some cases, to arise from insufficient understanding of the study and its rationale, which negatively impacted engagement with the study.

Teams who described having a strong sense of SIP ownership also highlighted that they experienced noticeable increases in their quality improvement efforts. The team manager of CRT 6 (which had the largest CRT fidelity scale increase), for example, appreciated that the SIP aimed to foster team ownership by offering the opportunity to focus improvement efforts on priorities that were important to staff.

*I think it was good that we were able to be really included in it, as well, and then actually do some of that work ourselves, sort of gave us more ownership, which was good. It’s really improved the morale, in the team, as well, as well as the figures and all that sort of stuff, too. (Staff CRT 6)*

Interwoven with CRT staff ownership and buy-in was staff motivation and morale, which was one of the most frequently cited process factor. A willingness and openness to change and engage with new ideas was associated with greater motivation and team morale, whereas staff cynicism, resistance to research, and a feeling that participation has been imposed from above, hindered engagement.

*I think it was more about people’s willingness and openness to change and for me I felt that the study, that the work done could have been more effective and it wasn’t as effective as it could have been because I don’t think the willingness was there, really. (Facilitator CRT 1)*.

A small number of staff members were largely unaware of the SIP, with a high rate of staff turnover making it difficult for SIP knowledge to be maintained and teams continuing to have buy in, and thus actions were not taken.

*I know there’s a big turnover of staff and I’ve only been here since May… more than half the team is gone and more than half the team is new. So I’m actually now one of the sort of longer stay people, but I think that we are just not… we just weren’t aware of it. And that’s nobody’s fault, it’s just time and pressure. (Staff CRT 2)*.

### SIP as a vehicle for change

The SIP was seen by many stakeholders as an important vehicle for change. Some stakeholders reported that through the fidelity review process, the SIP formalised existing plans for quality improvement work, and provided the time and opportunity to implement this: “*It does give you a framework and opportunity to learn from other people’s practice.”* (*Manager CRT 4*). The model increased awareness of best practice, enabled teams to reflect on their practice, and provided a framework to challenge and revise existing practices.

*A lot of the changes you would have wanted to have brought about anyway, but if you hadn’t have had the framework, you know, the time would have lapsed and lapsed and lapsed. (Staff CRT 5)*.

Related to this, stakeholders viewed the SIP as a means of benchmarking their practice through the fidelity review process. Several valued the SIP as it provided a quantifiable measure of practice, benchmarked at a national level that could evaluate and track their practice changes over time.

*Having outside people come in and analyse the work that you’re doing, that you trust, and knowing that there’s a national benchmark. And having us matched, or our work being matched nationally, so that we’re not going right off at a tangent and doing stupid things - that was really good. And I think having targets, because if people work to targets you have a sense of achievement. (Manager CRT 2)*.

### Culture of support from the top

Support from seniors in the Trust (the local healthcare organisation) was an important process factor. Motivation was higher in teams who felt supported by their Trust. This provided a sense of credibility to the SIP, and promoted engagement as it filtered down from management: “*We had the support of senior managers down the corridor. They were quite keen for us do well in the study and use it and improve the team.”* (*Manager CRT 6*).

*Senior management’s attitude across the Trust was really positive I felt and I didn’t realise how important that was when I started. So those meetings that you encouraged us to have just above the work were really important because I think every time things got difficult in the team I would go to those people in that meeting and ask them to kind of back it up really. So that was useful. (Facilitator CRT 3)*.

Conversely, CRTs that did not feel supported by the Trust described this as being demotivating and fostered a feeling that teams were acting alone in their work, making it difficult to affect change.

*It would have been more effective had there… as well as getting the teams involved, that there was actually buy-in from… at a more senior level within organisations and that was actually part of the agreement to work with those Trusts. (Facilitator CRT 1)*.

### The value of service user input

Where it took place, service user involvement was an important process factor. Stakeholders valued service user input, and highlighted that the SIP provided a platform for service user voices to be heard. Service user involvement was not achieved in all CRTs. Where it did take place, it was chiefly during service users presentations at the CORE events (scoping day and CORE learning events), service user participation in SIP working groups, and providing feedback to inform fidelity reviews.

*When we had our away day we had someone who’d used the service come and his mum, so they came to talk to us about their experience with being in a crisis and how, at that point he wasn’t under home treatment team, and how that affected them, not knowing how to get access. So we did some work with the CMHRS [community mental health team] around that. So that was really helpful, it was quite important really to have their experience and that on the back of an SI [serious incident] we’re more focused on carers views. (Manager CRT 1)*.

## Impact of the SIP

### Sustainable structural service improvements

A majority of stakeholders identified specific service improvement resulting from implementation of the SIP. These included: improved provision of psychological interventions, more systematic and detailed documentation processes, improved resources and hand-outs provided to service users, more time spent working with carers, and more work addressing physical health care needs. Related to this, several stakeholders mentioned ways in which greater engagement with service users had occurred following the SIP. This had enabled space to reflect on service user’s lived experiences and provided an opportunity to translate their suggestions into direct service improvement. Stakeholders described that interventions provided became more structured in nature, which improved the quality of the time spent with service users. Stakeholders from multiple teams described that a more holistic ethos to care had been adopted, and communication with service users such as notifying them about what time to expect their home visit, had also improved.

*Once he [service user worker] started working with us, the visits had been a problem, so we changed; we developed a pack as to what to expect and which we gave out to patients. And then we changed the timeframes of visits, so the clients could select what times they wanted, and that was better: contact improved dramatically, yes. (Manager CRT 2)*.

The majority of stakeholders wished for quality improvement work to remain ongoing after the study ended, attempting to fully embed changes made, and seek further feedback to ensure standards continue to improve:

*We want to keep it going. So I’ve got to work out a way, with my boss, as you might say, on how we can, kind of, embed it as an ongoing way of learning. (Manager CRT 3)*.

### Motivation and purpose

Stakeholders identified that the SIP improved the sense of team purpose, providing an opportunity to refocus, and address areas of practice that had previously been neglected: “*I think, as a whole, it got staff to reflect on what we do and why we do it.*” (*Manager CRT 3*). Linked to this, a majority of stakeholders highlighted that participating in the study helped them to see a bigger whole which fostered a sense of a collective identity.

*… [the team] gained a sense of being part of a larger collective enterprise. There are teams all around the country struggling with just what we do. (Facilitator CRT 4)*.

A positive impact on motivation was described at individual clinician and team levels, as well as on service users:

*That can-do attitude then spilled out to not just the work that the staff were doing in the team but the enthusiasm and the work that they were doing with the client group and enablement of that, of the client group, you know, spilled out. It’s the can-do attitude, and it’s quite empowering for patients, to have a clinician who enthuses them. (Manager CRT 2)*.

One facilitator described that beyond specific changes made, staff confidence in their ways of working had increased significantly following the SIP.

*The kind of confidence of the staff, the clarity in the care pathway, taking someone on, engaging with them and then discharging them in getting the right follow-up from their care coordinator in primary care, I imagine that kind of made a massive difference in terms of the experience for that service user of being in a crisis, coming to the crisis team, hopefully avoiding hospital admission and then going back to their care coordinator or their primary care. (Facilitator CRT 6)*.

### Team and inter-service communication

Many stakeholders suggested that the SIP improved within and between team communication. The new systems and processes adopted enabled staff to think and operate more consistently with each other. Several stakeholders noted that CRT communication and relationship with community and inpatient teams also improved as they had a better understanding of their role.

### A low level of SIP impact

Although less frequent, some staff described a low level or lack of impact of the SIP. For example, the manager from CRT 4, the service which demonstrated the smallest improvements in fidelity scores over the 12 months (+ 4), felt that, while useful as a general tool to help reflection, there had been no large changes in the service due to the SIP.

*I don’t think there’s been any major, you know, huge, landmark changes in the service that we provide, but it’s helped us to just look at how we can tweak things and make it just more relevant and helpful to the people that we’re providing a service for. (Manager CRT 4)*.

Numerous stakeholders expressed the view that it would take longer than the one-year study period to see results of new changes implemented by the SIP:

*If we’d had a bit longer we could have gradually… we were introducing a lot all at once I guess. We could have just eked it out a little bit maybe. (Facilitator CRT 3)*.

### Summary of between group commonalities and differences

Overall, there were some similarities and differences in opinion between managers, staff, and facilitators—with commonalities in opinion between groups being far more pronounced. Managers, staff, and facilitators all recognized the importance of a well-functioning CRT and the importance of team morale in this (with the SIP having positive impacts in this domain), the need for support from senior management in the CRT and the Trust, and the challenges of implementing changes, and making improvements, due to limited time and resources. All stakeholder groups spoke the usefulness of the CORE SIP (especially the fidelity resources), as it set clear expectations and standards, had a positive impact on clinical practice, and improved communication and collaboration.

The key differences in opinion between the three groups were that managers provided more mixed views on the effectiveness of the facilitator’s role than staff, with some seeing the facilitator role as vital and helpful, and others feeling there was a lack of engagement with the facilitator. Staff members were, in general, more positive about the facilitator’s role and the importance of this engagement. While all participant groups spoke of the importance of senior management, facilitators in particular spoke more frequently about the challenges of implementing service changes and placed more emphasis on the need for buy-in from influential and senior staff, as well as the challenges posed by staff turnover and geographical distance.

## Discussion

### Principal findings

Service improvement, especially at a national level, is challenging [15]. Despite this, the results of the SIP trial were promising: in teams receiving the SIP intervention, model fidelity rose in most intervention teams and was significantly higher than in control teams at follow-up; there were fewer in-patient admissions, lower in-patient bed use and better staff psychological health [15]. Our findings from the qualitative study presented above provide some insight into the complexities of implementing this type of intervention in CRT settings. Participating stakeholders in this study—including facilitators, CRT managers, and staff—outlined many positive impacts of the intervention, such as clarity of purpose, improved motivation and feeling ‘part of a bigger whole’, improved communication (with each other, and service users and carers), better understanding of good practice so sustained structural changes could be implemented. However, there were also considerable challenges, with lack of staff time being a key factor, and the necessity of creating a culture of service improvement through support from those in leadership positions.

Design of the CORE Service Improvement Programme was informed by the Evidence-Based Practice Program [18], which suggests the success of evidence-based service improvement projects depends on 5 key domains: (i) ensuring *Prioritisation* of the programme; (ii) *Leadership* support; (iii) *Workforce* development; (iv) *Workflow* re-engineering; and (v) practice *Reinforcement*. These domains are clearly observable in our findings, with *Leadership* being particularly impactful on the success of the SIP. Managers and senior staff within the Trust taking clear responsibility for leading the service improvement work, and engaging with the programme directly, was associated with higher satisfaction with the SIP and also better fidelity scores with the CRT. The importance placed on senior leadership in mental health services is also echoed elsewhere in the academic literature [22, 23].

*Workforce* re-engineering clearly required further consideration during the implementation. sufficient staffing was not consistent across CRTs, and this impacted the success of the SIP implementation. Ownership of the SIP by the workforce was key to effective implementation, with buy-in and a sense of choice in what they focused on being important for motivated engagement, rather than feeling as though the SIP was imposed on them. Where the SIP impact was viewed as weaker, high staff turnover and lack of time and resources were raised as key contributing factors. For example, due to high turnover in some CRTs, newer staff were unaware of the SIP as they had not been employed when training and the scoping day took place – they felt no ownership of improvement plans.

Having clear *Prioritization* of the SIP within CRTs mediated some of the workforce turnover issues in some of the CRTs. Specifically, CRT staff having a greater understanding of the programme through the SIP, fostered a sense of motivation in staff, and brought about a feeling of being part of a greater purpose as there was a connected collective across the UK working towards a broader and meaningful goal. The CRT fidelity scale and reviews offered as part of the SIP were seen as providing clear structures to monitor outcomes and provide feedback. In evidence-based practice literature, these initiatives sit under the *Reinforcement* domain. We cannot be certain it is the CRT fidelity scale that is the critical ingredient to service improvements: it is possible that improvements simply occur because the SIP provides CRTs with much needed time and space to think about how to improve their service. However, past evidence-based practice research suggests higher fidelity to evidence-based practices correlated with a more implementation activities occurring [18]. In this current study, it was the CRTs with higher fidelity that articulated the benefits of this *Reinforcement* via fidelity reviews more strongly. A review of evidence emphasised that fidelity measurement is essential to ensure that quality improvement interventions are delivered as intended and that the desired outcomes are achieved [24].

Some level of flexibility in terms of which SIP components CRTs implemented was necessary due to the enormous range of existing practices in CRTs [17], and local service contexts and team specific issues that change over time. Further, although the SIP was developed using evidence-based practice framework, it is clear that many of the barriers identified in this qualitative study related back to organisational readiness for change [25]. An a priori approach could have been applied to implementing change via the SIP. For example, alongside the benchmarking of the CRT via the initial fidelity reviews, stakeholders involved in implementing the new service improvement programs could collaboratively conduct an early assessment of their CRTs readiness to change before introducing new interventions. Other implementation science research in mental health setting has used the five evidence-based practice domains (prioritization, leadership, workforce, workflow, and reinforcement) for this very purpose [26]. This would allow identification of present barriers and provide implementers with the opportunity to address them. Services with less readiness for change may require more flexibility, or engagement with service leaders with the aim of addressing any remediable organisational barriers.

### Strengths and limitations

These qualitative findings provide important context and process-based understandings to the first RCT evaluating a service improvement programme with CRTs [15]. We interviewed a range of people involved in the intervention (staff, managers, facilitators) which offers diversity of perspectives. It may have been helpful to have interviewed senior Trust staff as well, in order to provide further insights to how Trusts can support such work in the future. It is likely, however, that the participants who were willing to be interviewed were those who had more positive things to say about the intervention or a greater sense of engagement and ownership. That said, there were a range of views expressed, both positive and negative, providing valuable insights to barriers and facilitators of the SIP. Similarly, the teams that agreed to participate in this interview study all increased their fidelity score during the intervention period, although the extent of improvement varied from just four to 37 points. Had we been able to interview staff from some of the teams that showed no improvement in fidelity score we may have heard different perspectives. Indeed, two initially selected CRTs decided not to participate in the qualitative feedback, so it is likely we received more positive feedback that we would have if these teams took part. An ameliorating factor is that we recruited the full cohort of facilitators. These facilitators all worked with more than one team, and nearly all worked with teams who increased fidelity score and teams who did not increase score, which has enabled us to capture the full range of teams to some extent.

The interviews were carried out by members of the research team known to most participants, due to involvement in the intervention over the 12-month period. This could have enabled interviewees to feel comfortable being open and honest, but could also have hindered such openness due to concerns that any negative comments might be disappointing to the interviewers. Similarly, analysis was carried out by some researchers who had spent considerable amounts of time with participating teams, and thus preconceived ideas may have influenced the themes considered salient (noting several authors had no direct involvement in SIP delivery). The research team attempted to counter any such influence by reflective note-keeping and explicit discussion of this issue. The interviewers were all white women in their late 20s and early 30s, which may have affected how comfortable interviewees felt sharing their experiences, as the interviewees were more diverse in terms of age and ethnicity. The wider research team included more diversity in terms of personal characteristics, which helped to provide different perspectives at the analysis stage.

### Research, policy, and practice implications and future directions

Further research with senior Trust managers could be beneficial in providing insights into how to ensure buy-in, and enable support of clinicians ‘on the ground’ to make meaningful improvements to their services. Similarly, work with policy-makers and those designing and managing acute care systems could help to embed the type of best practice benchmarks that all stakeholders in this study found so valuable.

In line with other organisational learning approaches in research [27], after receiving feedback on implementation we recommend additions or alterations to the CORE SIP. Our findings suggest that the following could be beneficial: conducting a readiness for change assessment and providing subsequent implementation support; providing routine top-up training and new staff onboarding; streamlining the fidelity review process; ensuring reviewers have a strong understanding of the CRT’s local context; finding ways to encourage more engagement with the online resources.

In terms of using this type of intervention with CRTs in the future, our findings suggest that there are some key points for stakeholders to consider. Firstly, the intervention worked well in teams where the manager and staff were engaged and motivated to implement the service improvement programme. Recent evidence suggests that ‘learning collaboratives’ (similar to the SIP learning events) can increase staff buy in, encourage healthy competition, and modelling of good practice [28]. Secondly, linked to the previous point, engagement was made much easier by senior Trust staff being visibly supportive of the study, which fed in to helping with issues around the lack of time, for example, in providing extra resources that enabled teams to carry out the work necessary. Thirdly, where service users and carers were involved in improvement work they added considerable value, with helpful and constructive input reported in every case. And fourthly, the use of the CRT fidelity scale as a model of best practice that teams could benchmark themselves against was fundamental in providing standards to aim for and see improvements against, as well as giving a sense of teams being part of a network of CRTs facing similar issues and sharing solutions. Finally, we know that training manuals/implementation guidance are not likely to be sufficient on their own to improve practice. Busy staff are unlikely to read such resources, let alone change their practice in response. Active leadership support, and coaching/field mentoring from a facilitator, opportunities for shared learning across teams, plus reinforcement by fidelity feedback are all crucial. The sustained support of facilitators in this study is consistent with evidence about the limitations of top down training in teams which are then left to attempt implementation on their own [29, 30].

The findings from this study show that stakeholders appreciated the flexibility and opportunities for ownership that the CORE SIP offered, as well as the sustained support from the facilitators and feedback from the fidelity reviews. In the future, a mixed methods study evaluating the impact of implementing the SIP should be conducted in routine care, rather than a trial context. This type of evaluation will help to validate and further refine these provisional ideas about programme theory. Further investigation is also needed to understand how coproduction with service users and carers can be incorporated into service planning and improvement, and particularly how the kind of local flexibility needed for such an approach can best fit with the kind of highly developed model outlined here.

Innovative, often unevaluated, models of community mental health crisis care have proliferated in recent years in England [4] and internationally [6]. It is important that these do not deflect attention from the need to improve quality of CRT care: CRTs remain a standard part of the English crisis care system [4] and implemented internationally; trial evidence suggests they can reduce hospital admissions and improve satisfaction with care [7, 31]. Yet they are typically implemented with low model fidelity [32, 33]. The CORE SIP is a programme which trial evidence shows can improve CRTs’ model fidelity and improve their effectiveness in reducing hospital admissions [15]: our paper provides important insights into how and why this is achieved, and critical ingredients for future service improvement initiatives.

## Conclusion

The qualitative views of CRT staff, managers, and facilitators analysed in this research have added depth and nuance to existing understanding about the challenges of implementing service improvement programmes. We found that critical ingredients to successful implementation include support from senior staff, team engagement and ownership, and sufficient resources to enable these things. But we also found that there were specific challenges encountered, and solutions found, in the different context in which participating teams were operating in. While fidelity measures provide useful benchmarks and standards for CRTs to aim for, the flexibility and opportunities to tailor the intervention outlined here were key in enabling and encouraging engagement and sustainability of service improvement activities.

### Electronic supplementary material

Below is the link to the electronic supplementary material.

Supplementary Material 1

## Data Availability

The datasets generated and/or analysed during the current study are not publicly available due to ethical restrictions on the sharing of interview transcript data, but may be available from the corresponding author on reasonable request.
