# Supplementary material for: Implementation of a crisis resolution team service improvement programme: a qualitative study of the critical ingredients for success
Source: Int J Ment Health Syst. 2024 May 4;18:18. doi: 10.1186/s13033-024-00638-6 (PMC11069280; doi:10.1186/s13033-024-00638-6)
Supplement: Supplementary file 1 — Supplementary Material 1 [file 13033_2024_638_MOESM1_ESM.docx]

# Appendix 1 – Topic Guide examples

**Topic guide for CRT staff focus groups/interviews**

*The CRT in which you work has been taking part in the CORE trial. This has involved testing out a service improvement programme over the last year. As part of the evaluation of this project, we would like to find out what staff working in CRTs which are taking part in the trial think of the resource kit.*

1. What do you think about the service improvement programme that has been tried out in your CRT?

2. What, if anything, was helpful about the programme?

3. What, if anything, was unhelpful about the programme?

*We’re interested to find out about the impact (if any) of the service improvement programme on clinical work and service delivery. For the next question, we’d like to ask you each in turn about any impact you think there has been on your individual clinical work. After that we’ll discuss as a group whether there have been any impacts on the CRT service overall.*

4. What impact, if any, has the programme had on your practice as an individual clinician or manager? *(Ask each participant in turn)*

5. What impact, if any, do you think the programme has had on the overall service delivered by your CRT?

6. What are your views about the CRT fidelity reviews?

- What was helpful about them?
- What was unhelpful about them?

7. What are your views about the Resource pack website?

- How, if at all, did you use the website during the year?
- What was helpful or unhelpful about it?

8. What are your views about the role of the Facilitator [insert name of facilitator here]?

- How was the facilitator helpful?
- How, if at all, was the facilitator unhelpful?

9. Were there any service users involved in any aspect of the service improvement programme?

10. What, if anything, prevented the programme from having an impact on the quality and development of services in your CRT?

11. Was there anything that hasn’t already been discussed that facilitated or helped the programme to have an impact on the quality and development of the CRT service?

12. Would you recommend the service improvement programme to other services?

- Why, or why not?

13. How could the programme be improved for future use?

14. Please tell us anything else you would like to say about the programme.

**Topic guide for interviews with Service Improvement Programme facilitators**

*In this interview, we would like to ask you about two things. First, we would like to hear about your experience of working in your facilitator role with your team(s). Next, we would like to hear your views about the CORE service improvement programme overall.*

**Part 1: local facilitator role**

1. Please confirm which CRT teams you acted as the Facilitator for. What was your connection to these teams and the local NHS Trust before and during the CRT resource kit programme?

2. Can you tell me about your experience of being the facilitator for this/these team(s)?

3. What aspects of your facilitator role do you think went well?

4. What aspects of your facilitator role do you think went less well?

*(Probe reasons)*

5. What has been the most challenging aspect of this role?

6. What obstacles to improving service quality did you encounter in your role?

*Prompt for impact of:*

- *Time available*
- *Resources/expertise available*
- *Attitude of CRT manager*
- *Attitude of CRT staff team*
- *Attitude or support from senior Trust management*

7. What factors, if any, helped you improve CRT service quality in your role?

8. What are your views about the training for your role provided by the CORE study team?

- What was helpful about it?
- What was unhelpful about it?
- How could it have been improved?

9. What are your views about the support from the CORE study team facilitator?

- What was helpful about it?
- What was unhelpful about it?
- How could it have been improved?

**Part 2: Views on CRT Service improvement programme**

10. What, if anything, was helpful about the service improvement programme overall?

11. What, if anything, was unhelpful about the service improvement programme overall?

12. What impact, if any, do you think the service improvement programme has had on the overall service delivered by your CRT?

13. What are your views about the CRT fidelity reviews?

- What was helpful about them?
- What was unhelpful about them?

14. What are your views about the Resource pack website?

- How, if at all, did you use the website during the year?
- What was helpful or unhelpful about it?

15. How could the service improvement programme be improved for future use?

16. Please tell us anything else you would like to say about the service improvement programme or your local facilitator role within this.

# Appendix 2

| **Team** | **Score change*** | **Fidelity items targeted** | **CRT description** | **CRT engagement** | **Facilitator summary** |
| --- | --- | --- | --- | --- | --- |
| CRT 1 | 105 to 123 (+18) | **8** Interface with community & EIP teams  **11, 13 & 14** Work with carers  **16** Monitoring & information on medication side effects  **17** Access to psychological therapies | Size: ~25 staff  Location: Based in a relatively affluent town in the south east of England, serving a semi-rural area | The manager was sceptical about the project, with engagement from the team challenging throughout the year. | The facilitator did much of the work themself and found it difficult to engage the team and keep track of the teams work. |
| CRT 2 | 98 to 111 (+13) | **2** Accessibility of CRT to eligible referrers  **7** Facilitating early discharge  **11** Comprehensive assessment  **24** Relapse prevention planning  **30** Staff induction & ongoing training  **36** Named worker system | Size: ~35 staff  Location: based in an economically deprived, urban area in Greater London. | The team had high staff turnover and was very busy. Communication with the manager was intermittent and many staff were not aware of the project. Some changes were introduced by the team’s psychologist without engagement from other staff. | The facilitator was minimally involved. Project meetings were infrequent, with no clear plan for quality improvement work established. |
| CRT 3 | 130 to 149 (+19) | **11** Assertive & comprehensive assessment  **14** Carer support  **16** Medication communication  **32** Lone working policy | Size: ~20 staff  Location: based in a historic seaside town on the south coast. | Due to the manager leaving, the project was initially met with some resistance from the team. Regular meetings with senior managers were required to promote engagement. Following this, staff followed project processes, with small working groups meeting regularly. | The facilitator was very enthusiastic and engaged, and visited the team at least fortnightly. Despite these efforts, the facilitator felt that the project was never fully embedded into the team. |
| CRT 4 | 138 to 142 (+4) | **11** Initial assessment  **12** Information for service users and families  **14** Work with carers  **24** Relapse prevention  **30** Supervision  **36** Named worker system | Size: ~35 staff  Location: based in an urban borough of London. | A working group and quality improvement plans were established early in the year. The team reported that they struggled to fully embed new quality improvement resources during the project, but were interested in continuing with this work following the end of the trial. | The facilitator was engaged and supportive, but did not visit the team more than once a month. |
| CRT 5 | 133 to 155 (+22) | **4 & 24** Planning for future crises  **6 & 7** Facilitating early discharge  **8 & 25** Aftercare & onward referral  **10** Distinct service  **12, 21 & 38** Timing, length & frequency of visit**s**  **13, 14 & 16** Support for service users & families  **17 & 29** Psychological interventions & staff to deliver  **22, 30, 31 & 32** Staff recruitment, induction, monitoring & trainin**g**  **34** Working with community services  **35** Equality & diversity  **36** Named worker system  **37** Alternatives to hospital | Size: ~25 staff  Location: based in a large town in the East Midlands. | The manager was extremely enthusiastic about the project. Some staff were very engaged, and overall the team followed project processes closely. The team was always busy, but made time for CORE. The facilitator reported work progressed albeit slowly during the project. | The facilitator was not based locally so visits to the team were infrequent with little staff interaction. |
| CRT 6 | 97 to 134 (+37) | **7** Facilitating early discharge  **8** Resource lending library  **15 & 16** Medication & prescribing  **20** CRT integrated care pathways  **27, 28, 29 & 30** Improve staffing levels  **30** Skill mapping and staff development  **34 & 35** Communication & boundaries | Size: ~20 staff  Location: based in a large town in Wiltshire. | At the beginning of the intervention period a new manager with a clear vision for improvement was appointed to the team. The new manager led the development of quality improvement plans enthusiastically, with monthly updating meetings taking place. Levels of staff engagement varied over the course of the project. | Facilitator visits to the team were infrequent. |
